# Associations between Proprioceptive Neural Pathway Structural Connectivity and Balance in People with Multiple Sclerosis

**DOI:** 10.3389/fnhum.2014.00814

**Published:** 2014-10-20

**Authors:** Brett W. Fling, Geetanjali Gera Dutta, Heather Schlueter, Michelle H. Cameron, Fay B. Horak

**Affiliations:** ^1^Department of Neurology, School of Medicine, Oregon Health & Science University, Portland, OR, USA; ^2^Portland VA Medical Center, Portland, OR, USA

**Keywords:** somatosensory cortex, somatosensory disorders, white matter pathways, diffusion tensor imaging, diffusion tensor tractography, proprioception

## Abstract

Mobility and balance impairments are a hallmark of multiple sclerosis (MS), affecting nearly half of patients at presentation and resulting in decreased activity and participation, falls, injuries, and reduced quality of life. A growing body of work suggests that balance impairments in people with mild MS are primarily the result of deficits in proprioception, the ability to determine body position in space in the absence of vision. A better understanding of the pathophysiology of balance disturbances in MS is needed to develop evidence-based rehabilitation approaches. The purpose of the current study was to (1) map the cortical proprioceptive pathway *in vivo* using diffusion-weighted imaging and (2) assess associations between proprioceptive pathway white matter microstructural integrity and performance on clinical and behavioral balance tasks. We hypothesized that people with MS (PwMS) would have reduced integrity of cerebral proprioceptive pathways, and that reduced white matter microstructure within these tracts would be strongly related to proprioceptive-based balance deficits. We found poorer balance control on proprioceptive-based tasks and reduced white matter microstructural integrity of the cortical proprioceptive tracts in PwMS compared with age-matched healthy controls (HC). Microstructural integrity of this pathway in the right hemisphere was also strongly associated with proprioceptive-based balance control in PwMS and controls. Conversely, while white matter integrity of the right hemisphere’s proprioceptive pathway was significantly correlated with overall balance performance in HC, there was no such relationship in PwMS. These results augment existing literature suggesting that balance control in PwMS may become more dependent upon (1) cerebellar-regulated proprioceptive control, (2) the vestibular system, and/or (3) the visual system.

## Introduction

Multiple sclerosis (MS) is the most common chronic, non-traumatic neurological disorder of young adults, affecting approximately 400,000 people in the United States (Zwibel, 2009). The ability to maintain one’s balance while standing is a fundamental aspect of human behavior that is known to deteriorate in the presence of nervous system disease. Balance deficiencies are an early hallmark of MS and are common in individuals with minimal (Nelson and Anderson, 1995; Corradini et al., 1997; Karst et al., 2005; Martin et al., 2006) or even no clinically assessable impairments (Daley and Swank, 1981), becoming more pronounced in those with significant disease advancement (Jackson et al., 1995; Soyuer et al., 2006). Mobility impairments are also identified as the primary concern in maintaining functional independence and quality of life in people with MS (PwMS) and are associated with a multitude of adverse outcomes including falls, injury, reduced activity, and mortality (Zwibel, 2009; Sutliff, 2010).

Proprioceptive (somatosensory) feedback, principally supplied by primary muscle spindles (Ia), is critically important for balance control. Disrupting proprioception during standing increases body sway (Inglis et al., 1994; Lajoie et al., 1996; Horak et al., 2002), a well-accepted indicator of postural stability. For example, patients with peripheral neuropathy affecting 1a and 1b afferents show large body sway with eyes closed and delayed postural response latencies (Dickstein et al., 2001). It is worth noting that functional alterations in smaller fibers such as group II spindle afferents have also related to postural unsteadiness (Nardone et al., 2000). Studies comparing balance control under different sensory feedback conditions (e.g., eyes closed, standing on a compliant surface, etc.) estimate that proprioception contributes 58–69% to body sway in standing (Lord et al., 1991). Ankle joint proprioception appears to be of particular importance, as it provides the most salient information regarding standing body sway (Fitzpatrick et al., 1994) and is a sensory feedback system significantly degraded in PwMS (Horak et al., 1990; Wylezinska et al., 2003; Cameron et al., 2008).

People with MS have a decreased ability to maintain static postural position (Daley and Swank, 1981, 1983; Corradini et al., 1997; Soyuer et al., 2006; Chung et al., 2008; Cattaneo and Jonsdottir, 2009), and their postural sway increases disproportionately with their eyes closed compared to control participants (Daley and Swank, 1981; Corradini et al., 1997). In addition, proprioceptively triggered postural responses to external perturbations are significantly delayed in PwMS (Cameron et al., 2008), thus this somatosensory feedback potentially reaches the brain too late for appropriate motor corrections. This is supported by work demonstrating that beyond fundamental peripheral reflex mechanisms, central processing of proprioceptive signals from the foot/ankle is critical for balance control.

Increased neural activation in proprioceptive-processing neural areas is associated with better proprioceptive-based balance performance (Goble et al., 2011, 2012). Goble et al. (2012) utilized fMRI to determine the neural components of proprioception (i.e., brain activity), demonstrating neural activity in the primary sensorimotor cortex, thalamus, and basal ganglia in young and older adults when high frequency vibration devices stimulated proprioception-related receptors in the feet. The authors also reported an association between the amount of neural activity in the somatosensory cortices and the ability of both young and older adults to perform an ankle position-matching test and eyes-closed standing sway assessment (Goble et al., 2011). These results support an association between proprioceptive-related functional brain activity and balance, but the structural connectivity of proprioceptive pathways has not been studied in healthy or clinical populations. It is unlikely that dysfunction occurs in the proprioceptive receptors (e.g., muscle spindles) in PwMS (Feys et al., 2006), but rather in the transmission and processing of sensory feedback due to damaged white matter pathways restricting sensory signal conduction. A number of studies have reported associations between white matter microstructure and upper extremity function in PwMS (Lowe et al., 2006; Bonzano et al., 2008, 2011, 2014), but little is known about the impact of white matter disruption on control of the lower limbs. Further, fiber tracking of specific proprioceptive pathways has yet to be undertaken in the human brain.

A better understanding of the pathophysiology of balance disturbances in MS is needed to develop evidence-based rehabilitation approaches. Successful rehabilitation of balance disorders in PwMS requires clinicians to identify the balance deficits so that approaches to retraining can be specific and effective (Boyd et al., 2007; Alexander et al., 2009). The purpose of the current study was to (1) map the cortical proprioceptive pathway *in vivo* using diffusion-weighted imaging and (2) assess correlations between proprioceptive pathway white matter microstructural integrity and performance on clinical and behavioral balance tasks. We hypothesized that PwMS would have reduced integrity of proprioceptive pathways in the brain, and that reduced white matter microstructure within these tracts would be strongly related to proprioceptive-based balance deficits.

## Materials and Methods

### Participants

Twenty-five people with mild to moderate MS (21 women and 4 men) were recruited through the Multiple Sclerosis Center of Oregon clinic at Oregon Health & Science University (OHSU). Twenty age-matched healthy controls (HC) were also recruited from the surrounding Portland, OR area. Participants were excluded if they could not safely walk 20 feet without walking aids, had a joint replacement, musculoskeletal or vestibular disorder, dementia, claustrophobia, severe tremor, or had metal in their body. The Expanded Disability Status Scale (EDSS) was utilized to assess clinical disability in PwMS; all participants had to have a value less than 4.0 indicating the individual was fully ambulatory without aid and could walk at least 500 m without aid. OHSU’s Institutional Review Board approved this study, and all participants gave their informed written consent prior to beginning the experiment.

### Balance assessments

**Figure 1:**
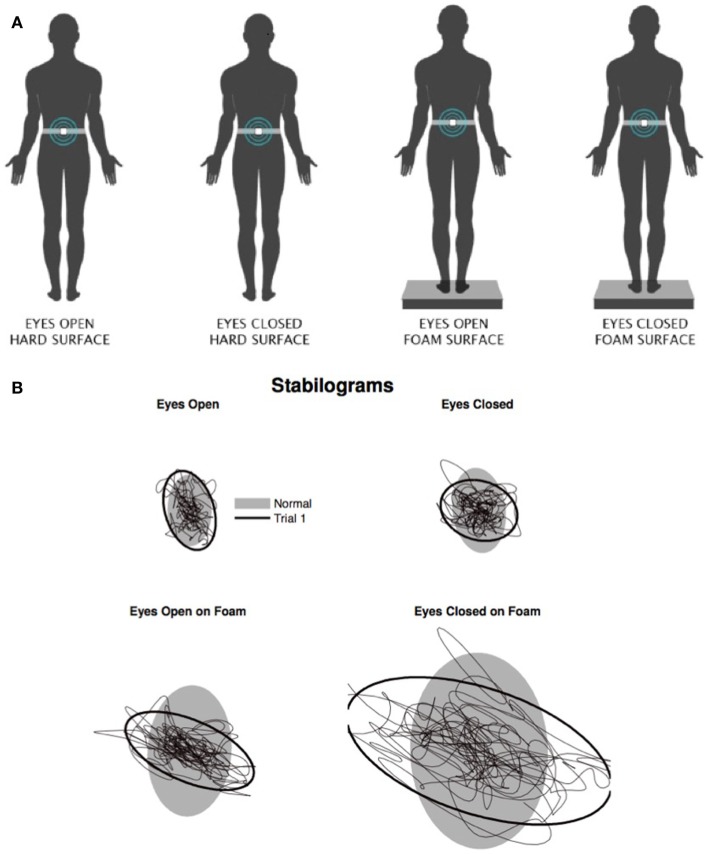
**The four conditions used for the instrumented clinical test of sensory integration and balance (ICTSIB) (A)**. Representative postural sway plots from a PwMS **(B)**.

Dynamic gait and balance were assessed using the mini balance evaluation systems test (Mini-BESTest), a brief clinical rating scale with strong psychometric characteristics that assesses four distinct domains: anticipatory postural adjustments, reactive postural control, sensory orientation, and balance during gait (Franchignoni et al., 2010). Additionally, quiet stance under differing sensory conditions was characterized using the clinical test of sensory interaction for balance (CTSIB). The CTSIB systematically tests the influence of proprioceptive (somatosensory), vestibular, and visual inputs on standing balance control (Shumway-Cook and Horak, 1986). The current study solely utilized conditions 1, 2, 4, and 5, thus removing the conditions where visual feedback is altered (Figures 1A,B**)**. This test can be instrumented (ICTSIB) by using a single body-worn accelerometer to provide objective and practical measures of postural control (Mancini et al., 2012). In the current study, we used a wireless, inertial Opal sensor (APDM Inc., USA) composed of a 3-D accelerometer, a 3-D gyroscope, and a 3-D magnetometer. The sensor was positioned with an elastic belt on the posterior trunk, near the body’s center of mass, and data were acquired and automatically analyzed with MobilityLab (Mancini and Horak, 2010). The ICTSIB was chosen because it is a rapid, clinical assessment of sensory contributions to balance control and induces minimal fatigue. Additionally, multiple rest breaks were provided over the course of testing to mitigate the potential effects of fatigue.

We also calculated the Romberg quotient for total sway area: Romberg quotient = total sway eyes closed ÷ total sway eyes open, for both the firm- and foam-surface conditions. For the firm-surface conditions, the Romberg quotient is reflective of proprioceptive and vestibular contributions to balance, whereas the Romberg quotient for the foam-surface conditions solely reflects vestibular contributions to balance (Shumway-Cook and Horak, 1986).

### Image acquisition

On another day, separated by less than 2 weeks, participants were scanned on a 3.0 T Siemens Magentom Tim Trio scanner with a 12-channel head coil at OHSU’s Advanced Imaging Research Center. One high-resolution T1-weighted MPRAGE sequence (orientation = Sagittal, echo time = 3.58 ms, repetition time = 2300 ms, 256 × 256 matrix, resolution 1.0 mm × 1.0 mm × 1.1 mm. total scan time = 9 min 14 s) was acquired. A whole-brain echoplanar imaging sequence was used (TR = 9,100 ms, TE = 88 ms, field of view = 240 mm^2^, *b* value = 1,000 s/mm^2^, isotropic voxel dimensions = 2 mm^3^); images were sensitized for diffusion along 90 different directions with a *b* value of 1000 s/mm^2^. For every 36 diffusion-weighted images, a non-diffusion-weighted image (*b* = 0 s/mm^2^) was acquired (three total). A static magnetic field map was also acquired using the same parameters as the diffusion-weighted sequence. All neuroimaging testing occurred in the morning to maintain homogeneity of testing across participants.

### Diffusion tensor imaging analysis

Diffusion data were processed using the tools implemented in FMRIB Software Library (FSL; Version 5.0). The three raw datasets were first corrected for eddy current distortions and motion artifacts using FMRIB’s diffusion toolbox (FDT 1.0), then averaged to improve signal-to-noise ratio (Eickhoff et al., 2010) and subsequently skull-stripped (using FSL’s brain extraction tool). The principal diffusion direction was estimated for each voxel as a probability density function, using Bayes’ rules in order to account for noise and uncertainty in the measured data. As described elsewhere (Behrens et al., 2003), the implicit modeling of noise in a probabilistic model enables a fiber tracking procedure without externally added constraints such as fractional anisotropy (FA) threshold or fiber angle. Thus, fiber tracking in or near cortical areas becomes more sensitive. For each individual, the FA images were normalized into Montreal Neurological Institute (MNI) space by using a linear (affine) registration and Fourier interpolation through the FMRIB linear image registration tool. Using the averaged images with *b* = 0 and *b* = 1000 s/mm^2^, the diffusion tensor was calculated. Diagonalization of the diffusion tensor yields the eigenvalues λ1, λ2, and λ3, as well as the eigenvectors that define the predominant diffusion direction. Radial diffusivity (RD), an indirect neural marker of myelination (Gulani et al., 2001), was calculated for each participant by taking the mean of the second and third eigenvalues – (λ2 + λ3)/2. Lower RD is interpreted as being indicative of better white matter tract microstructure (Beaulieu, 2011). We also provide FA values, a more general measure of white matter microstructure, where higher values are indicative of better overall tract quality.

### Region of interest selection

Often termed “conscious” proprioceptive feedback, the cortical proprioceptive tract ascends the large myelinated fibers of the dorsal column–medial lemniscus pathway and is responsible for transmitting fine touch, vibration, and conscious proprioceptive information from the body to the cerebral cortex (Gardner and Jessell, 2000; Johnson et al., 2008). Within the brain, this sensory feedback initially synapses in the ipsilateral gracile nucleus (GN), prior to decussating and ascending the medial lemniscus pathway to the contralateral ventral posterolateral (VPL) nucleus of the thalamus and terminating in the primary somatosensory cortex (S1) (Gardner and Jessell, 2000). All regions of interest (ROIs) were created in MNI 1 mm space and then affine-transformed into each participant’s native diffusion space to carry out tractography.

#### S1 ROIs

In both the right and left hemisphere, we created a hand-drawn mask encompassing the anterior portion of the post-central gyrus. The masks were constrained to solely include gray matter via a gray matter segmentation template and further restricted from crossing the central sulcus anteriorly via a pre-central gyrus exclusion mask. *Z* range: 94–145.

#### VPL ROIs

In both the right and left hemisphere, we utilized a conversion of the original Talairach structural labeling, a digitized version of the original (coarsely sliced) Talairach atlas (Lancaster et al., 2000) after the application of a correcting affine transform (Lancaster et al., 2007) to register it into MNI152 space to identify the VPL nucleus.

#### GN ROIs

In both the right and left hemisphere, we created a hand-drawn mask using previously identified coordinates (*X*: −4/+4, *Y* : −44, *Z*: −56; zu Eulenburg et al., 2010). Each mask encompassed 18 total voxels.

#### Brodmann area 3a ROIs

In both the right and left hemisphere, we utilized a conversion of the original Talairach structural labeling, a digitized version of the original (coarsely sliced) Talairach atlas (Lancaster et al., 2000), after the application of a correcting affine transform (Lancaster et al., 2007) to register it into MNI152 space to identify Brodmann area 3a (BA 3a).

The proprioceptive pathway was identified within each hemisphere using (1) the S1 as the seed mask, (2) the ipsilateral VPL as a waypoint mask, and (3) the contralateral GN as the termination mask. In addition, we performed separate analyses for tracts within this pathway that originate/terminate in Brodmann area 3a, as opposed to the entire S1 ROI, as this is the principal cortical region where proprioceptive feedback is processed (Gardner and Jessell, 2000). Probabilistic fiber tracking [using FDT 1.0; see Behrens et al. (2007)] was initiated from every voxel within the binarized seed ROI in each participant’s native diffusion space. Streamline samples (25,000) were sent out from each voxel, with a step length of 0.5 mm and a curvature threshold of 0.2. For group analyses, the probabilistic connectivity distribution maps from individual participants were thresholded at 50% (thus selecting all connections where >12,500 of 25,000 samples passed); a very conservative level in comparison to previous work using a threshold of 5% (Gschwind et al., 2012; Fling et al., 2013). Tracts were then binarized and affine-transformed with tri-linear interpolation into MNI space and summed across participants to obtain the connectivity probability maps of the group. RD was calculated for (1) all tracts identified within the pathway and (2) just those tracts within the pathway that terminated/originated in BA 3a (as opposed to the entire S1 ROI). FA was also calculated for all tracts identified within the cortical proprioceptive pathway.

### Statistical analysis

All statistical analyses were carried out using SPSS (IBM SPSS Statistics v21). Unless otherwise noted, a *P*-value ≤0.05 was considered significant. Homogeneity of variance was assessed with a Levene’s test of equality of error variances, no differences were noted between groups (*P* > 0.1), and thus parametric analyses were utilized. We conducted one-tailed independent groups *t*-tests to test the hypothesis that PwMS would perform worse than HC participants on the Mini-BESTest. In addition, we performed a 2 × 4 (group × condition) repeated measures analysis of variance (RMANOVA) for sway area derived from the ICTSIB. Significant main effects were subjected to *post hoc* analyses using independent *t*-tests and Bonferroni-corrected for multiple comparisons. Group differences in the Romberg Quotient were assessed by a one-tailed *t-*test and Bonferroni-corrected for multiple comparisons (α = 0.05/2). Whole-brain group differences in white matter microstructural integrity were assessed with tract-based spatial statistics (TBSS). RD images were used as input for TBSS (Smith et al., 2006). This was accomplished by registering all subjects’ FA images to a common space (the FA158 MNI space template) using a combination of affine and non-linear registration, creating the mean FA image, eroding it to a skeleton, and thresholding the skeleton at FA > 0.25. The resulting alignment-invariant representation of the central trajectory of white matter pathways was used for voxelwise statistical analysis (randomized, 10,000 permutations). The contrast PwMS < HC was examined using threshold-free cluster enhancement (TFCE) (Smith et al., 2007), with correction for multiple comparisons at α < 0.01. Microstructural integrity of fiber tracts comprising the cortical proprioceptive pathway were compared between groups using a 2 × 2 (group × hemisphere) RMANOVA to test the hypothesis that PwMS have poorer cortical proprioceptive tract microstructural integrity. A similar comparison was performed for tracts solely terminating/originating within BA 3a. Significant fiber tract main effects were further analyzed by independent *t-*tests and corrected for multiple comparisons. Finally, linear regression analyses was performed to assess correlations between proprioceptive tract microstructure and balance performance assessed by (1) total Mini-BESTest and the (2) Romberg quotient calculated from total sway area for the firm-surface and foam-surface conditions of the ICTSIB and were corrected for multiple comparisons (α = 0.05/3). Five control participants were unable to return for balance testing, thus we are presenting neuroimaging results from 20 HC, however, only provide data regarding balance control within 15 participants. Moreover, two PwMS were unable to maintain static balance control during the eyes-closed conditions of the ICTSIB (for both firm- and foam-surface conditions); therefore, their data are not included for those sway conditions or for the Romberg quotient measures. Data are reported as mean **±** standard deviation (SD) unless otherwise indicated.

## Results

Demographic and clinical characteristics of the participants can be seen in Table 1. Briefly, participants were well matched for age and weekly physical activity; however, the PwMS group had significantly more females compared to the HC group (χ^2^ = 4.5; *P* < 0.05).

**Table 1:** **Demographics and clinical characteristics of study participants**.

	Healthy controls (*N* = 20)	PwMS (*N* = 25)
Age	51.0 (3.18)	48.3 (2.51)
Physical activity (h/week)	4.9 (0.67)	4.8 (0.65)
Gender (M/F)	**9/11**	**4/21**
Mini-BESTest (Max = 28)	**25.9 (0.47)**	**20.4 (1.26)**
EDSS	N/A	3.5 (0.19)
Disease duration (years)	N/A	12.2 (1–26)

*Group differences are highlighted in bold text. Data are mean (± SEM), with the exception of disease duration, which is presented as mean (range)*.

### Balance results

**Figure 2:**
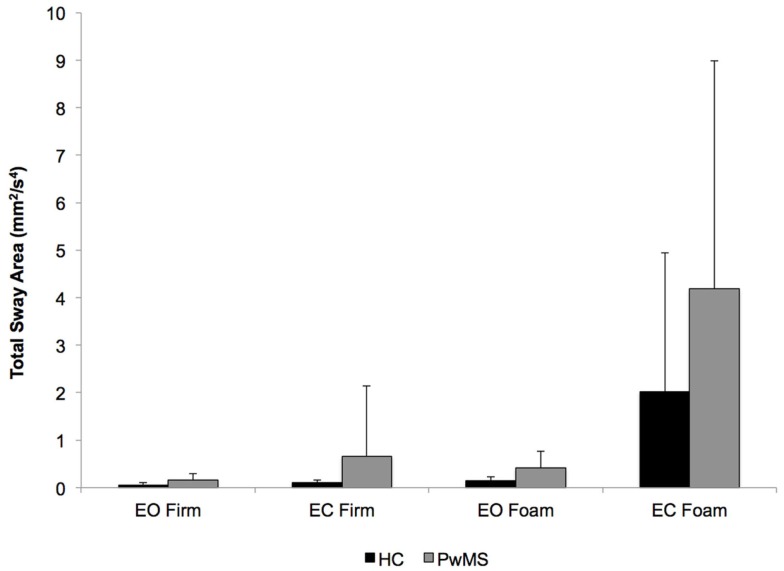
**Total sway area for each of the four conditions tested with the ICTSIB**. A significant main effect of group (*P* < 0.05), but lack of a group × condition interaction, is reflective of PwMS having greater sway area for all conditions compared to HC. Data are mean (± SD).

As expected, PwMS had significantly poorer dynamic balance control (*t* = 3.3; *P* < 0.001) than HC as assessed by the Mini-BESTest (Table 1). Particular deficits in PwMS were observed within the anticipatory postural adjustment, sensory orientation, and dynamic gait domains, whereas reactive postural control performance was quite similar between groups. Data obtained during the ICTSIB revealed significant group (*F*_1,35_ = 3.9; *P* < 0.05) and condition (*F*_1,35_ = 14.3; *P* < 0.001) main effects for total sway, but no group × condition interaction (*F*_1,35_ = 2.6; *P* < 0.12) likely due to the large variability in both groups on the eyes-closed conditions (Figure 2). For all conditions, sway area was greater in PwMS, indicative of decreased static balance control. In addition, PwMS had a higher Romberg quotient for both the firm- (PwMS: 2.9; HC: 2.0) and foam-surface conditions (PwMS: 10.4; HC: 9.7), but these differences were not statistically significant (firm surface: *P* < 0.06; foam surface: *P* < 0.3).

### Imaging results

**Figure 3:**
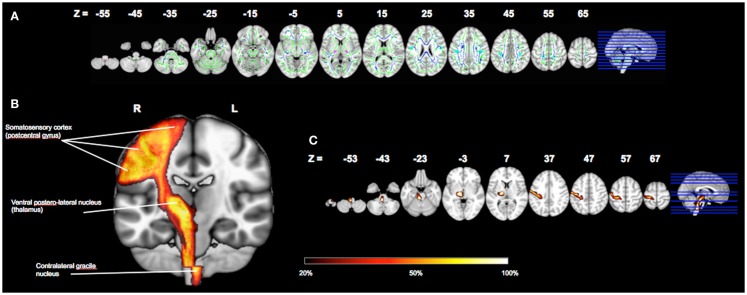
**Whole brain voxelwise tract-based spatial statistical (TBSS) analysis of radial diffusivity (A)**. Voxels with significantly poorer white matter quality in PwMS are shown in blue (TFCE, multiple comparison corrected; *P* < 0.01) and are overlaid on the TBSS skeleton (green). Bilateral Brodmann area 3a is highlighted in light blue (*Z* = 25–55), the ventral posterolateral nuclei of the thalamus in pink (*Z* = 5) and the gracile nuclei in red (*Z* = −55). Data are displayed in MNI space and radiologic convention. The right hemisphere’s cortical proprioceptive pathway identified within all study participants [**(B,C)**; left hemisphere not shown]. The color bar indicates the percentage of participants with identified tracts in each region. All data are displayed in MNI space and radiologic convention.

**Figure 4:**
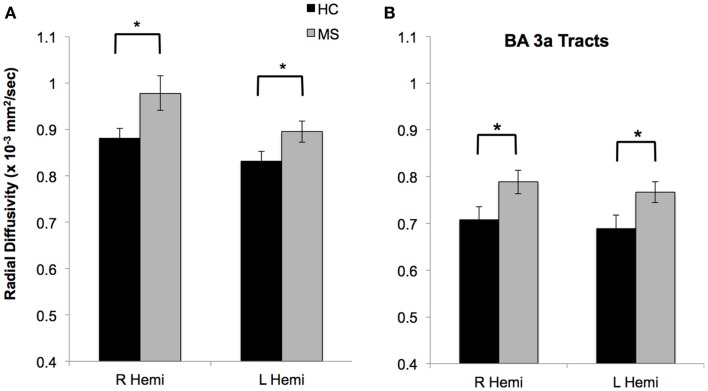
**Microstructural integrity of the entire cortical proprioceptive fiber tracts (A) and fiber tracts of the same pathway restricted to solely those that originate/terminate in Brodmann area 3a (B)**. For both plots, PwMS have significantly greater radial diffusivity of tracts within the right and left hemispheres compared to HC. Data are mean (± SD); *P < 0.05.

Whole-brain voxelwise tract (TBSS) analysis of RD revealed significant differences between groups in multiple regions. In particular, PwMS had significantly higher RD principally within periventricular regions, corticospinal, and callosal fiber tracts (Figure 3A**)**. The cortical proprioceptive tract was identified in all participants within both the right and left hemispheres (Figures 3B,C). When computed for the entire cortical proprioceptive tract, no group differences were observed (*P* > 0.3) for FA values in either the right (PwMS = 0.30 ± 0.02; HC = 0.32 ± 0.03) or left hemispheres (PwMS = 0.33 ± 0.03; HC = 0.34 ± 0.02). Conversely, we report a significant main effect of group such that PwMS had significantly greater RD (*F*_1,43_ = 4.3; *P* < 0.05) compared to HC. There was no group × hemisphere interaction (*F*_1,43_ = 0.4; *P* > 0.5), indicating poorer tract integrity in PwMS in both the right (PwMS = 0.96 × 10^−3^ mm^2^/s ± 0.15: HC = 0.88 × 10^−3^ mm^2^/s ± 0.09) and left hemispheres (PwMS = 0.89 × 10^−3^ mm^2^/s ± 0.11: HC = 0.83 × 10^−3^ mm^2^/s ± 0.09) (Figure 4A). Additionally, we found a main effect of hemisphere (*F*_1,43_ = 19.0; *P* < 0.001) such that fiber tracts within the right hemisphere had significantly greater RD (0.92 × 10^−3^ mm^2^/s ± 0.13) than the left (0.86 × 10^−3^ mm^2^/s ± 0.11).

A significant group difference was also found when restricting cortical proprioceptive tracts to only those that originate/terminate in Brodmann area 3a (*F*_1,43_ = 7.2; *P* < 0.05; Figure 4B), again demonstrating poorer tract integrity in PwMS compared to HC. For these tracts, no hemispheric differences were noted (*F*_1,43_ = 0.1; *P* > 0.7), nor was there a significant group × hemisphere interaction (*F*_1,43_ = 0.05; *P* > 0.8).

### Associations between balance and proprioceptive tract integrity

**Figure 5:**
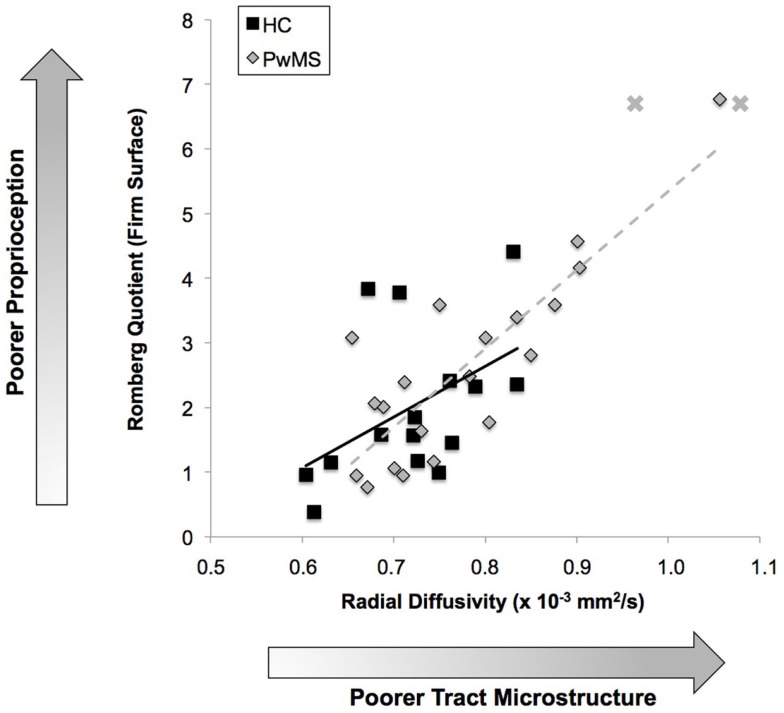
**Relationship between proprioceptive pathway integrity and proprioceptive-based balance**. Poorer integrity of the right hemisphere’s proprioceptive tracts originating/terminating in BA 3a is associated with poorer proprioceptive-based balance control. Two PwMS were unable to complete balance trials with eyes closed, thus their data are visualized as X’s on the figure with their Romberg quotient represented as the maximum value observed in the PwMS group. These two datapoints are not included in the linear regression model. HC: *r* = 0.47; *P* < 0.07; PwMS: *r* = 0.74;*P* < 0.001.

Poorer integrity of the right hemisphere’s cortical proprioceptive pathway (higher RD) was associated with lower Mini-BESTest scores in HC (*r* = −0.58; *P* < 0.03; not significant when corrected for multiple comparisons), but not in PwMS (*r* = −0.31; *P* < 0.27; Table 2) suggesting that proprioceptive feedback is utilized to a lesser degree in PwMS for overall balance control. Similarly, we report a strong correlation between the Romberg quotient (foam standing) and fiber tract integrity of the right hemisphere’s entire cortical proprioceptive pathway in HC (*r* = 0.61; *P* < 0.02), but not in PwMS (*r* = 0.11). This suggests that in HC, vestibular components of balance may also be related to processing and neural conduction within fiber tracts projecting to/from the primary somatosensory cortex, although this requires further inquiry. Poorer integrity of the right hemisphere’s fiber tracts restricted to BA 3a was significantly associated with poorer proprioceptive-based balance control as assessed by the Romberg quotient derived from the firm-surface conditions in PwMS (*r* = 0.74, *P* < 0.001) and to a lesser extent in HC (*r* = 0.47, *P* < 0.07; Figure 5). This significant relationship is unaffected in PwMS when removing the individual with the worst balance control and fiber tract microstructure (*r* = 0.73, *P* < 0.001), and appear to be further strengthened when considering the two PwMS who were unable to maintain eyes-closed balance also possessed very poor fiber tract microstructure (see Figure 5). These results demonstrate that white matter integrity of pathways transmitting proprioceptive feedback strongly influenced somatosensory-based balance control in HC and, to an even greater extent, PwMS. Finally, no associations were observed between metrics of balance performance and proprioceptive tract integrity within the left hemisphere for either group.

**Table 2:** **Correlation coefficients (*r*) from linear regression between balance measures and proprioceptive tract radial diffusivity**.

	Mini-BESTest		Romberg quotient: firm surface		Romberg quotient: foam surface	
	HC	PwMS	HC	PwMS	HC	PwMS
Cortical proprio tract – R Hemi	−0.58*	**−**0.31	0.33	0.24	0.61*	0.11
Cortical proprio tract – L Hemi	**−**0.44	**−**0.33	0.14	**−**0.16	0.22	**−**0.12
BA 3a proprio tract – R Hemi	**−**0.50	**−**0.32	0.47	0.74**	0.33	0.27
BA 3a proprio tract – L Hemi	**−**0.38	**−**0.32	0.08	0.03	0.22	0.12

*P < 0.05; **P < 0.001.

## Discussion

To our knowledge, this is the first study to utilize diffusion imaging to identify white matter tracts comprising the cortical proprioceptive pathways within the human brain. As hypothesized, PwMS had reduced white matter integrity of the entire cortical proprioceptive tract and those fibers that originate/terminate in BA 3a. Furthermore, microstructural integrity of this pathway (solely within the right hemisphere) was strongly related to proprioceptive-based balance control in both PwMS and age-matched control participants. Conversely, while white matter integrity of the entire proprioceptive pathway was significantly correlated with overall balance performance in HC, this relationship was mitigated in PwMS. We speculate that balance control in PwMS may become more dependent upon (1) cerebellar-regulated proprioceptive control, (2) the vestibular system, and/or (3) the visual system.

Balance impairments are an early hallmark of PwMS and are common in individuals with minimal or even no clinically assessable impairments, becoming more pronounced in those with significant disease progression (Karst et al., 2005). Postural control of balance requires appropriate sensory integration of visual, somatosensory, and vestibular inputs. A limited body of literature indicates balance deficits in PwMS when all sensory systems are available (quiet standing with eyes open), suggesting involvement of vestibular and visual inputs (Williams et al., 1997; Cattaneo and Jonsdottir, 2009; Van Emmerik et al., 2010). Nonetheless, instability in PwMS is particularly evident in the absence of vision suggesting that instability is largely the result of deficits in proprioceptive feedback (Daley and Swank, 1981; Rougier et al., 2007). Data from the current study are consistent with these findings; PwMS had reduced postural balance control across all conditions, as well as a differential increase in postural sway when standing on a firm surface with their eyes closed. Many PwMS have delayed and hypermetric automatic postural responses to recover balance in response to surface perturbations (Cameron et al., 2008). Furthermore, delayed proprioceptive conduction in PwMS is related to reduced postural stability (Cameron et al., 2008). Due to slow spinal somatosensory conduction, it is likely that PwMS receive delayed and/or distorted proprioceptive feedback of postural displacements and thus compensatory postural adjustments are late, incorrect, or absent.

While Goble et al. (2011) previously reported proprioceptive-related brain activity is strongly related to balance in the absence of vision, no studies have assessed structural connectivity of cortical proprioceptive pathways in either HC or PwMS. Microstructural integrity of white matter pathways can be significantly degraded in PwMS, impairing the propagation of neural signals. Typical white matter in PwMS results in decreased FA and increased RD (Werring et al., 1999; Bammer et al., 2000; Ciccarelli et al., 2001; Filippi, 2001), with the latter a reflection of decreased myelination (Gulani et al., 2001). We report PwMS had reduced white matter integrity throughout multiple regions in the brain. Of particular relevance to postural balance control, PwMS had reduced white matter integrity of the entire cortical proprioceptive pathway and of fiber tracts in the pathway that originated/terminated in BA 3a. Proprioceptive information from the lower limbs that reaches the primary somatosensory cortex is transmitted up the spinal cord in the dorsal columns and synapses within the ipsilateral GN prior to decussating and ascending via the medial lemniscus to the ventral posterolateral nuclei of the thalamus and are relayed on to the post-central gyrus, specifically Brodmann area 3a (Johnson et al., 2008). The pathway deficits reported in the current cohort of mildly affected PwMS echo recent work demonstrating significantly reduced volume of the thalamus in PwMS early in the disease (Henry et al., 2008). Functionally, the human thalamus is a complex brain relay center responsible for both sensory and motor functions (Percheron et al., 1996). Recent work has demonstrated that activation of the thalamus during standing is critical to maintain balance and avoid falls (Zwergal et al., 2011). Moreover, thalamic atrophy is associated with a wide range of clinical manifestations including cognitive decline, motor deficits, fatigue, and ocular motility disturbance in PwMS (Minagar et al., 2013).

A large body of literature demonstrates associations between white matter integrity and motor performance of the upper extremities (Gooijers and Swinnen, 2014), but similar approaches with regard to the lower limbs have received limited attention. Van Impe et al. (2012) report minimal correlation between white matter microstructure and proprioceptive/vestibular-based balance control in healthy young and older adults. Prosperini et al. (2013) report that balance impairment was correlated with poorer white matter integrity along the cerebellar connections and supratentorial associative white matter bundles in PwMS. In individuals with non-specific low back pain, decreased integrity of the superior cerebellar peduncle was associated with an increased reliance on ankle muscle proprioception – indicating an impaired proprioceptive weighting capacity (Pijnenburg et al., 2014). In the current study, we found that white matter integrity of BA 3a tracts was strongly related to proprioceptive-based balance in PwMS. This relationship was similar, although not significant in HC, potentially due to a limited behavioral range on the relatively easy static balance tasks used in the current study. Furthermore, white matter integrity of the entire proprioceptive pathway was significantly correlated with overall balance control in HC; however, this relationship was mitigated in PwMS. A delay in proprioceptive input from the feet/ankles results in late postural responses as well as inaccurate sensory feedback about the postural responses necessary for motor learning and may significantly reduce the potential for navigation and adaptation within dynamic walking environments (Rosenkranz and Rothwell, 2012). Due to the long response-loop necessary for proprioceptive feedback from the ankles to reach the somatosensory cortex, be centrally processed, and send an efferent response back to the muscle, PwMS may rely less upon cortically mediated proprioceptive feedback. While this hypothesis requires further evaluation, we speculate that balance control in PwMS may become more dependent upon (1) cerebellar-regulated proprioceptive control, (2) the vestibular system, and/or (3) the visual system. For example, a promising study in a moderately sized cohort of PwMS demonstrated significant and clinically relevant improvements in balance control following vestibular rehabilitation (Hebert et al., 2011).

Finally, it is important to note that all observed associations between proprioceptive balance control and fiber tract structure in the current study were restricted to the right hemisphere. Lateralization of mechanisms mediating functions such as inhibition (Aron, 2007), language (Hutsler and Galuske, 2003), and perception (Hutsler and Galuske, 2003) is widely accepted as a fundamental feature of neural organization. This literature has extended into the motor control of upper limbs in recent years revealing that each brain hemisphere contributes unique control mechanisms to the movements of each arm (Mutha et al., 2012). These studies suggest a greater relative reliance of the left hemisphere on visual feedback for movement, while the right hemisphere is more adept at processing proprioceptive information [for a thorough review, we direct the reader to Goble and Brown (2008)]. There is currently a dearth of literature regarding the cortical mechanisms underlying lower limb control; however, a limited body of work supports a dominant role of the right hemisphere in gait and balance control. Dating back to the seminal work of Geschwind (1965), left hemispheric lesions are often associated with bilateral limb apraxia, whereas whole body movements are largely unaffected, indicating an increased role of the right hemisphere in these movements. Following stroke, right hemiparetics (in whom the right hemisphere was uninjured) progressed more rapidly and achieved higher levels of functional mobility following gait training (Cassvan et al., 1976). Further, Rapcsak et al. (1993) demonstrated that the right hemisphere is preferentially involved in programing and executing familiar, automated movements (e.g., locomotion). These findings are supported by work demonstrating a right hemisphere dominance in body schema and spatial orientation (Wolpert et al., 1998). The results of the current study fit well with the theory of right hemisphere dominance for processing proprioceptive feedback, from both the upper (Goble et al., 2012) and now the lower limbs.

There are limitations to the current study. Differences observed in either balance or neuroimaging measures could be attributed to gender differences between groups in the current study. Wolfson et al. (1994) report no differences in quiescent stance between males and females. Furthermore, fatigue is a well-known component of reduced motor performance in PwMS, and leads to an increased probability of falls (Finlayson et al., 2006). The ICTSIB was chosen for this study because it is designed for a rapid, clinical assessment of balance. While we made efforts to reduce fatigue in our participants, it is possible that differences in perceived exhaustion and exertion may contribute to the observed deficits in balance control. It has also been suggested that reduced sensorimotor control in PwMS could be the result of an increasingly sedentary lifestyle, secondary to the disease, rather than the impairments caused by the disease itself (Ng et al., 2004). The participants in this study were all mildly affected by their disease and maintained a relatively active lifestyle with common activities including swimming, biking, ice skating, Tai Chi, and yoga. Thus, it is doubtful that daily activity levels underlie the differences observed in the current study. A contemporary study also reports greater fiber tract microstructure in the splenium of the corpus callosum in women (Kanaan et al., 2014) assessed via TBSS like in the current study. The authors also report increased integrity of the superior cerebellar peduncles in males (Kanaan et al., 2014). As none of the fiber tracts identified in our cortical proprioceptive pathway traverse either of these areas, we feel it is unlikely to influence our diffusion imaging results. A noticeable challenge to the current imaging approach was to identify fiber tracts that decussate within the brain stem. It is possible that some fiber tracts were unable to be appropriately tracked; however, we utilized a 2-fiber model (Behrens et al., 2007) to mitigate this issue along with a conservative fiber tracking threshold to ensure strong consistency within and across individuals.

## Conclusion

Balance and locomotion deficits are ubiquitous in PwMS, particularly in the absence of vision. Consistent with previous literature, we report reduced proprioceptive-based balance in PwMS. Furthermore, this study details a novel methodology using diffusion tensor imaging to identify the cortical proprioceptive tracts in the human brain. Fiber tract integrity of this pathway was significantly poorer in PwMS and was strongly related to proprioceptive balance control in both healthy participants and PwMS. On the other hand, while white matter integrity of the proprioceptive pathway was significantly correlated with dynamic balance control in HC, this association was mitigated in PwMS. These results augment existing literature suggesting that balance control in PwMS may become more dependent upon (1) cerebellar-regulated proprioceptive control, (2) the vestibular system, and/or (3) the visual system. Future studies investigating the potential compensatory nature of these additional sensory systems for balance control in PwMS will further our understanding of the sensory integration and potential re-weighting necessary to maintain upright balance (Assländer and Peterka, 2014).

## Conflict of Interest Statement

The Oregon Health & Science University and Dr. Horak have a significant financial interest in APDM, a company that may have a commercial interest in the results of this research and technology. This potential institutional and individual conflict has been reviewed and managed by OHSU.
